# Understanding the rise in traditional contraceptive methods use in Uttar Pradesh, India

**DOI:** 10.1186/s12978-022-01547-y

**Published:** 2023-01-06

**Authors:** Vasanthakumar Namasivayam, Bidyadhar Dehury, Ravi Prakash, Marissa Becker, Preeti Anand, Ashish Mishra, Shreya Singhal, Shivalingappa Halli, James Blanchard, Dean Spears, Shajy Isac

**Affiliations:** 1 Institute for Global Public Health, University of Manitoba, R070 Med Rehab Bldg, 771 McDermot Avenue, Winnipeg, MB R3E 0T6 Canada; 2 India Health Action Trust, Lucknow, India; 3 Department of Economics and Population Research Center, University of Texas at Austin, TX Austin, USA

**Keywords:** Traditional methods, Initial contraceptive method, Consistent use, FP method availability, Uttar Pradesh

## Abstract

**Background:**

The sustainable development goals (SDG) aim at satisfying three-fourths of family planning needs through modern contraceptive methods by 2030. However, the traditional methods (TM) of family planning use are on the rise, along with modern contraception in Uttar Pradesh (UP), the most populous Indian state. This study attempts to explore the dynamics of rising TM use in the state.

**Methods:**

We used a state representative cross-sectional survey conducted among 12,200 Currently Married Women (CMW) aged 15–49 years during December 2020–February 2021 in UP. Using a multistage sampling technique, 508 primary sampling units (PSU) were selected. These PSU were ASHA areas in rural settings and Census Enumeration Blocks in urban settings. About 27 households from each PSU were randomly selected. All the eligible women within the selected households were interviewed. The survey also included the nearest public health facilities to understand the availability of family planning methods. Univariate and bivariate analyses were conducted. Appropriate sampling weights were applied.

**Results:**

Overall, 33.9% of CMW were using any modern methods and 23.7% any TM (Rhythm and withdrawal) at the time of survey. The results show that while the modern method use has increased by 2.2 percentage points, the TM use increased by 9.9 percentage points compared to NFHS-4 (2015–16). The use of TM was almost same across women of different socio-demographic characteristics. Of 2921 current TM users, 80.7% started with TM and 78.3% expressed to continue with the same in future. No side effects (56.9%), easy to use (41.7%) and no cost incurred (38.0%) were the main reasons for the continuation of TM. TM use increased despite a significant increase (66.1 to 81.3%) in the availability of modern reversible methods and consistent availability of limiting methods (84.0%) in the nearest public health facilities.

**Conclusion:**

Initial contraceptive method was found to have significant implications for current contraceptive method choice and future preferences. Program should reach young and zero-parity women with modern method choices by leveraging front-line workers in rural UP. Community and facility platforms can also be engaged in providing modern method choices to women of other parities to increase modern contraceptive use further to achieve the SDG goals.

**Supplementary Information:**

The online version contains supplementary material available at 10.1186/s12978-022-01547-y.

## Plain language summary

In Uttar Pradesh, the use of traditional methods of contraception is on the rise, observed similarly in many other Indian states in recent times. The emphasis on modern contraceptive methods and the rise and high prevalence of traditional method use in the state call for a systematic assessment to understand the dynamics such as patterns, prevalence and reasons for traditional method use for better family planning programming. Using a state representative cross-sectional survey data from Uttar Pradesh, we attempted to understand the dynamics of increasing traditional methods use. We found no significant variations in use of traditional methods by their socio-demographic characteristics. Not only that, most current traditional method users reported that their first method was a traditional method and an overwhelming proportion of women (4/5 traditional methods users) expressed to continue with the same method in future. Also the findings reveal that more than half of the traditional method users used the method consistently over the three-years calendar period. Among those who had unmet need at the time of survey, a considerable proportion of them intend to use traditional methods in future. This emphasized the importance of initial contraceptive method choice on current contraceptive use and future preference. Traditional methods use increased in the state despite a significant increase (66.1 to 81.3% during 2018 to 2021) in availability of modern reversible methods and consistent availability of limiting method (84.0%) in public health facilities.

**Supplementary Information:**

The online version contains supplementary material available at 10.1186/s12978-022-01547-y.

## Background

In 1952, India became the first country to adopt a national Family Planning Program (FPP) with the objective of “reducing birth rate to the extent necessary to stabilize the population at a level consistent with the requirement of national economy” [1]. Over the years, India’s family planning program has undergone different strategic shifts in terms of policy and program implementation. Initially, it was a “clinic approach” which assumed that those who need family planning would visit such clinics without any hesitation and subsequently modified to the cafeteria approach with a basket of choices in the 1960s followed by massive sterilization drive through a camp approach by the government in mid-1970s [2]. Following the International Conference on Population and Development (ICPD) in 1994, rather than only focusing on fertility regulations, sexual and reproductive health and rights of women in the reproductive age (aged 15–49) were prioritized [2]. Later, India aligned with the “Millennium Development Goals (MDG) target 5B” to provide universal access to reproductive health by 2015 [3]. Currently, the Sustainable Development Goals (SDG) indicator 3.7 adopted by India aims at ensuring universal access to sexual and reproductive health care services by 2030 by satisfying three-fourth of family planning needs through modern contraceptive methods [4].

Uttar Pradesh (UP) is the most populous state of India with an estimated population of 233 million [5] accounting for 17% of India’s population with a wide district-level heterogeneity in socio-economic status of its population. During the period 1992–2021, the unmet need for family planning among Currently Married Women (CMW) in UP reduced from 30.1 to 12.9% and the overall demand met with any contraceptive method among CMW increased from 39.7 to 82.9%. However, the increase in demand met by modern contraceptive methods didn’t increase at the same pace [6, 7]. Specifically, the overall demand satisfied by any contraceptive method in the state increased by 43 percentage points whereas the demand met by modern contraceptive methods increased by only 22 percentage points (37.1 to 59.1%) between 1992 and 2021 [6, 7]. Within the modern contraceptive methods, during 2015–2021, the use of sterilization remained more or less the same among CMW (around 17.0%), use of other reversible methods (oral pills, injectable and IUDs) together hovered around 5–6%, and the use of condom increased substantially from 10.8 to 19.1% [7, 8].

In contrast to the modern contraceptive methods, the use of traditional methods (TM) in UP (mainly rhythm method and withdrawal) increased from 1.3% in 1992 to 13.8% in 2015 which further increased to 17.9% in 2021 [6–8]. Interestingly, in 21 out of 37 states and union territories of India, over 10% of CMW in the reproductive age group reported using a traditional method of contraception in 2019–21 [9, 10]. The emphasis on modern contraceptive methods in the SDGs and the rise and high prevalence of TM use in UP call for a systematic assessment to understand the dynamics such as patterns, prevalence and reasons for TM use etc. for better family planning programming in UP to achieve the SDG goal.

Despite the higher level of TM use in UP, only a few studies [11, 12] have attempted to understand the paradox of faster increase in TM use compared to that of modern contraceptive methods. While the National Family Health Surveys (NFHS) could help in understanding the overall traditional contraceptive prevalence and user profile of a state, it has limitations to understand more nuanced details of TM use such as initial methods, future intention, program contact coverage, availability of modern family planning methods, correct knowledge of contraceptive methods, etc. The Uttar Pradesh Technical Support Unit (UPTSU) established by the University of Manitoba (UoM) in collaboration with the India Health Action Trust (IHAT) is supporting the Government of Uttar Pradesh (GoUP) in its’ efforts to address the family planning (FP) unmet needs of couples by expanding the availability, quality and voluntary uptake of modern contraceptive methods across all the 75 districts of the state. UPTSU conducted a population-based survey to understand the contraceptive use patterns and dynamics and to assess programmatic progress. Using this data, we focused on understanding the contraceptive use dynamics among TM users in comparison to modern contraceptive users. In addition, the contraceptive use behaviour and future intention to use contraceptives among CMW, who expressed an unmet need, have also been analyzed to develop a better understanding to further support the GoUP’s goal of reducing unmet need for family planning. The present research is expected to offer new insights on TM users and the way forward for UP’s family planning program in the context of SDG 2030.

## Methods

### Study setting

A state representative cross-sectional survey was conducted in UP during December 2020–February 2021. This was an integrated survey in which data from households with women in the reproductive age range 15–49 years were linked to data from care providers from public facilities of the catchment area of Primary Sampling Unit (PSU) as well as data from community-level front line workers (FLWs) such as Accredited Social Health Activist (ASHA) and Auxiliary Nurse Midwives (ANM). UP has 18 administrative divisions, 75 districts, 820 blocks and more than 97,000 inhabited villages making it a large and complex setting to implement any public health program due to the diversity in the culture as well as socio-demographic and economic composition of the population.

### Sample size and sampling design

The sample size for this study was determined using the divisional-level modern contraceptive prevalence rate (mCPR) of NFHS-4 (2015–16). The primary respondents for the study were CMW aged 15–49 years and a total of 12,700 CMW were required for the study. The required sample size in each division was proportionally allocated to both rural and urban areas in proportion to the population distribution within the division as per Census of India 2011. Altogether, 508 PSUs (394 rural and 114 urban) were selected across the state. Within each division, a two-stage sampling technique was adopted. In the first stage, the required number of PSU were selected using the Probability Proportional to Size (PPS) approach using a list of habituated census villages in rural areas and Census Enumeration Blocks (CEBs) in urban areas. The area covered by an ASHA, who is a female health activist responsible for providing outreach health-related services to the catchment area with a population of about 1000–1500 individuals across 250–300 households [13], was considered as the PSU in rural areas. If the selected village covered less than 300 households in the ASHA area, then all the households in that PSU were listed. However, in any selected PSU with more than 300 households and which was served by more than one ASHA, then one ASHA catchment area was selected randomly. Further, if there were more than 300 households served by only one ASHA in the selected village, then segments of 150–200 households were made and a maximum of two segments were selected using PPS. The same approach of segmentation was adopted in urban areas if there were more than 300 households in the selected CEB. In the second stage, approximately, 27 households in each PSU were randomly selected using a systematic random sampling approach. A household listing was done in all selected PSUs before the main survey which provided a sampling frame for the selection of households. In total 12,200 CMW participated in the study and were interviewed with a response rate of 96%.

In addition, 496 public health facilities (Primary Healthcare Centres and higher-level facilities) catering to the selected PSU were selected for a facility readiness assessment for FP services. One doctor and one staff nurse, usually engaged in providing FP services in the selected facilities, were also interviewed. A total of 476 doctors and 451 staff nurses were included in the study. The study interviewed all the available FP counsellors (n = 223) in the state. The study also interviewed FLWs-419 ASHAs and 370 ANMs serving the selected rural PSUs providing community-based FP services. Also, the study observed  Village Health and Nutrition Day (VHND) in half of the selected rural PSUs. VHND is a community-based platform where outreach services like routine immunization, antenatal care, and family planning services are provided by ANM in an ASHA catchment area once a month.

Written consent was obtained from all adult participants. Assent was taken from the CMW aged 15–17 years with written consent from their husband/head of the household. All consenting CMW aged 15–49 years within a household, who stayed in the selected household on the night before the survey, were interviewed. Written consent was obtained either from the chief medical superintendent / chief medical officer / medical officer-in-charge in the selected facilities before observing the facility. Written consent was also obtained from the care providers who participated in the study.

### Survey questionnaire

A household questionnaire was administered to an adult member or selected women participant in the households selected under the study. Socio-economic information about the household along with the details of family members were captured in the household questionnaire. Women's questionnaire was administered to all CMW from the selected household to capture information on their demographic characteristics, reproduction, marriage and cohabitation, contraception use, fertility preferences, and program exposure. In addition, domestic violence, decision making, spousal communication on FP, self-efficacy related to FP, myths and misconceptions related to contraceptive methods, and mass media exposure were captured using globally validated standard tools [8, 14–16]. A contraceptive and fertility event calendar that captured month by month history of contraceptive and reproductive events including live birth, stillbirth, miscarriage and abortion for the 36 months calendar period preceding to survey (starting from January 2018) was also administered. The contraceptive and fertility event calendar also covered information on the source of obtaining the contraceptive methods or source of services for reproductive events and reasons for discontinuing contraceptive methods.

Trained female research investigators administered the questionnaire in the local language (*Hindi*). Handheld mobile devices with Open Data Kit (ODK) based (Android) applications were used for data collection. In addition, as part of the quality assurance mechanism, female supervisors were appointed to monitor and supervise the fieldwork, including daily spot/back-check of interviews.

### Measures

#### Outcome

We measured the current contraceptive method used through the question “Are you (or your husband) currently doing something or using any method to delay or avoid getting pregnant?”. If responded ‘no’ then they were considered as nonusers and those who responded ‘yes’ were then asked, “which method(s) are you currently using?”. Response options were female sterilization, male sterilization, IUCD- Copper-T/ Loop, depot medroxy progesterone acetate (“*Antara”)*, oral contraceptives including levonorgestrel & ethinyloestradiol, and ormeloxifene (also known as Centchroman or “*Chhaya*”), levonorgestrel (known as emergency contraception), male condom, female condom, lactational amenorrhea method, other modern methods (MM) such as diaphragm, foam or jelly, and traditional methods including rhythm method, withdrawal and other traditional methods. The CMW were classified into three groups based on the current use of contraceptive methods: MM users (female sterilization, male sterilization, IUCD- Copper-T/ Loop, *Antara*, oral contraceptive pills, *Chhaya*/ Centchroman, emergency contraception pills, male condom, female condom, lactational amenorrhea method, other modern methods), traditional method users (rhythm/withdrawal /other traditional methods) and non-users. The respondents were also asked about their initial contraceptive method use by asking “Which method did you first use to delay or avoid getting pregnant?”. The CMW were also classified as those who started with any modern method, started with a traditional method and those who never used any method. We computed unmet need for family planning as the percentage of currently married women who either want to space their next birth or stop childbearing entirely but are not using contraception according to the revised definition of unmet need as described in Demography Health Surveys (DHS) globally [17].

#### Independent variables

The analysis included residence (rural, urban), caste group (Scheduled Caste (SC)/Scheduled Tribe (ST), Other Backward Castes (OBC) and others), religion (Hindu, non-Hindu), wealth quintile (poorest, poor, middle, rich and richest), age of the respondent (15–24, 25–29, 30–34, 35–39, and 40–49), parity (0, 1, 2, 3, and 4 +), education of the respondent and husband (< 5 years of schooling, 5–9 years of schooling, 10–11 years of schooling and 12 years of schooling or more), husband’s occupation (non-agricultural labour, salaried, cultivator/ agricultural labour, business and others), husband’s frequency of home visit (lives at home, visit home once in 1–3 months, visit home once in 4–6 months and visit home once in a year or later), fertility preferences (no more child, wanted to have another child within 2 years and wanted to have another child after 2 years), attainment of desired sex composition of the children (no sex preference, didn’t achieve desired composition for either sex, achieved desired number of boys but not girls, achieved desired number of girls but not boys, achieved desired sex composition), and FLWs interaction (frequency and discussion on FP). In addition, correct knowledge of modern reversible methods and traditional methods, reasons for continuing the method, and sources of information on the traditional methods were also included in the analysis. Definitions used to determine correct knowledge of MM and TM are provided in Additional file 1: Table S1.

### Analysis

Descriptive analyses such as percent distribution, mean, standard deviation (SD), and median with interquartile range were used to describe the sample characteristics. The bivariate analysis presented the association of independent variables and the use of contraceptive methods. A comparison was made in the initiation, continuation and switching of methods and future preferences among TM and MM users. Consistency/switching in the use of TM and modern reversible methods were analyzed using the 3-year contraceptive and fertility event calendar data among a cohort of TM users (n = 2796) and modern reversible methods users (n = 1406) at the beginning of the calendar month and presented using Sankey diagram. Appropriate sampling weights were used in all the analyses except for Sankey diagram and availability of family planning methods in public health facilities. The "don’t know" response and missing data were considered as a separate group while describing sample distribution. However, these two categories were not included in the bivariate tables. There were 41 (0.3%) cases for which caste, religion, and household wealth quintile information were missing and 15 (0.1%) cases of "don’t know" category in the caste variable. Preferred timing of next birth was missing for 165 (1.6%) cases. All analyses were conducted using STATA 16 [18].

## Results

### Profile of the respondents

Of the 12,200 CMW aged 15–49 years enrolled in the study, 77.1% belonged to rural areas while the remaining 22.9% to urban areas (Table 1). Nearly 53.6% of women were from the OBC category followed by 29.1% in the lowest social group (SC/ST) and 16.8% in the other category. The majority of the CMW were Hindu (84.3%), the median age of CMW was 32 years (IQR: 25–40), and an average of around 3 children per CMW (mean number of children ever born: 2.9 (SD 1.9)), with 9.9% at parity 0, 14.5% at parity 1 and 31.6% with parity 4 or more. The mean years of schooling of the CMW was 5.7 years (SD 5.7) with 44.3% having less than 5 years of schooling and another 30.3% having more than 10 years of schooling. The mean years of schooling of the participant’s husband was 8.4 years (SD 8.3). Husband’s occupation was reported as 41% engaged in a non-agricultural occupation, followed by cultivation/ agricultural labour (20.6%), salaried job (19.6%) and business (16%). About 20% of respondents reported that their husbands were migrants, mostly short-term migrants (13.7%) and the remaining six percent were long-term migrants with at least 6 months of migration. Two-thirds of the CMW did not want any more children, 12.2% wanted to have another child within two years and 12.6% wanted to have children after two years. About 18.4% of CMW stated no sex preference and 38.6% had achieved the stated preferred sex composition of their children. About 16% had achieved the desired number of boys but not girls and 18.9% had achieved the desired number of girls but not boys. Nearly two-thirds of CMW ever had any contact with FLWs, 43.1% had contact in the last three years and, among them, 31.6% were informed about FP methods.

**Table 1:** Percent distribution of currently married women aged 15–49 years, Uttar Pradesh

Background characteristics	Overall (n = 12,200)
% Belonged to urban residence	22.9
% Belonged to SC/ST households	29.1
% Belonged to OBC households	53.6
% Belonged to Hindu religion	84.3
Age	
% Aged below 25 years	20.5
% Aged 25–29 years	20.5
% Aged 30–34 years	17.8
% Aged 35–39 years	15.5
% Aged 40–49 years	25.7
Median age, years (IQR)	32 (25, 40)
Mean number of children ever born (SD)	2.9 (1.9)
Parity	
% With 0 parity	9.9
% With 1 parity	14.5
% With 2 parity	23.4
% With 3 parity	20.6
% With parity 4 +	31.6
Education	
% With < 5 years of schooling	44.3
% With 5–9 years of schooling	25.4
% With 10–11 years of schooling	7.9
% With 12 + years of schooling	22.4
Mean years of schooling of women (SD)	5.7 (5.7)
Mean years of schooling of husband (SD)	8.4 (8.3)
Husband’s occupation	
% Non-agricultural labour	41.0
% Salaried	19.6
% Cultivator/ agricultural labour	20.6
% Business	16.0
% Other	2.9
Husband's frequency of visits to home	
% Lives at home	80.3
% Visit home once in 1–3 months	7.5
% Visit home once in 4–6 months	6.2
% Visit home once or less in a year	6.0
Fertility preference	
% Want to have another child within 2 years	12.2
% Want to have another child after 2 years	12.6
% Want no more child	68.0
% Reported can't get pregnant	5.7
% Undecided or missing information	1.5
Mean ideal number of children (SD)	2.5 (0.95)
Desired sex composition	
% Stated no sex preference	18.4
% Didn't achieve either sex	8.5
% Had achieved desired number of boys, but not girls	15.5
% Had achieved desired number of girls, but not boys	18.9
% Had achieved desired sex composition	38.6
% Ever had any contact with FLWs	66.5
% Had any contact with FLWs in last 3 years	43.1
% Were informed by FLWs about FP methods among those who had interacted with FLWs in last 3 years (n = 5469)	31.6

### Socio-demographic differentials in contraceptive use

Overall, 53.7% of CMW reported ever use of any modern method and 57.7% ever used a traditional method (Table 2). Results further indicated that 57.6% CMW were using any contraceptive method at the time of the survey; 33.9% were modern contraceptive users, and 23.7% used any traditional methods (rhythm or withdrawal). Among MM users, 17.4% were sterilized, 12.6% were using condoms and the remainder 3.9% were using other modern contraceptive methods. Among the TM users, 19.4% were using rhythm method, 4.2% were using withdrawal method and 0.1% were using other traditional methods. The mCPR was higher among the CMW who belonged to urban areas (40.5% versus 31.9% in rural areas), Hindu women (35.6% versus 24.4% among non-Hindu), those belonging to the richest quintile (41.4%), and who belonged to the higher age groups, notably among those 30–34 years (40.9%) and 35–39 years (42.3%). Similarly, the mCPR was highest at 44.4% among those with parity 3 followed by 37.1% among those with parity 4 and more.

**Table 2:** Percentage of CMW ever used any modern method, traditional methods and distribution of current users by method mix according to their socio-demographics

Characteristics	Ever used MM [95% CI]	Ever used TM [95% CI]	CPR [95% CI]	mCPR [95% CI]	TM [95% CI]	N
Overall	53.7 [52.8–54.6]	57.7 [56.8–58.5]	57.6 [56.7–58.4]	33.9 [33.1–34.7]	**23.7** [22.9–24.4]	12,200
Place of residence						
Rural	50.9 [49.9–51.9]	58.6 [57.6–59.6]	56.1 [55.1–57.1]	31.9 [31.0–32.9]	**24.2** [23.3–25.1]	9,704
Urban	63.2 [61.4–65.0]	54.6 [52.7–56.4]	62.4 [60.5–64.1]	40.5 [38.7–42.3]	**21.9** [20.4–23.4]	2,496
Caste						
SC/ST	53.0 [51.3–54.6]	59.2 [57.6–60.8]	57.6 [55.9–59.1]	34.7 [33.1–36.3]	**22.9** [21.5–24.3]	3,665
OBC	52.6 [51.4–53.8]	58.2 [57.0–59.4]	57.7 [56.5–58.9]	32.9 [31.8–34.1]	**24.8** [23.7–25.8]	6,438
Others	58.7 [56.5–60.8]	53.6 [51.5–55.8]	57.4 [55.2–59.5]	35.8 [33.7–37.8]	**21.6** [19.9–23.5]	2,041
Religion						
Hindu	55.1 [54.2–56.1]	58.3 [57.4–59.3]	58.8 [57.9–59.8]	35.6 [34.7–36.6]	**23.2** [22.4–24.1]	10,369
Non-Hindu	46.1 [43.8–48.3]	54.2 [51.9–56.4]	50.4 [48.1–52.6]	24.4 [22.5–26.4]	**26.0** [24.1–28.0]	1831
Wealth quintile						
Poorest	47.0 [44.9–49.2]	58.6 [56.5–60.7]	54.3 [52.2–56.4]	30.4 [28.5–32.4]	**23.9** [22.1–25.8]	2028
Poor	47.7 [45.7–49.8]	59.4 [57.4–61.4]	54.8 [52.8–56.8]	31.6 [29.8–33.5]	**23.2** [21.5–24.9]	2457
Middle	51.3 [49.4–53.2]	57.9 [56.0–59.8]	56.5 [54.6–58.4]	32.0 [30.3–33.9]	**24.5** [22.8–26.1]	2622
Rich	57.1 [55.2–59.0]	57.2 [55.3–59.1]	57.9 [56.0–59.8]	33.8 [32.0–35.6]	**24.1** [22.5–25.8]	2609
Richest	64.3 [62.4–66.2]	55.5 [53.5–57.5]	64.1 [62.1–65.9]	41.4 [39.4–43.4]	**22.7** [21.0–24.4]	2443
Current age						
15–24	35.7 [33.8–37.6]	43.1 [41.2–45.1]	37.5 [35.7–39.4]	18.1 [16.7–19.7]	**19.4** [17.9–21.0]	2556
25–29	57.5 [55.6–59.4]	58.9 [56.9–60.8]	59.8 [58.0–61.8]	33.6 [31.8–35.5]	**26.2** [24.6–28.0]	2508
30–34	60.1 [58.0–62.1]	62.5 [60.5–64.5]	69.2 [67.2–71.1]	40.9 [38.9–43.0]	**28.3** [26.4–30.2]	2145
35–39	60.9 [58.7–63.1]	61.1 [58.9–63.3]	71.6 [69.6–73.6]	42.3 [40.1–44.6]	**29.3** [27.3–31.4]	1875
40–49	56.3 [54.6–58.1]	62.9 [61.2–64.6]	55.1 [53.4–56.9]	36.7 [35.0–38.4]	**18.4** [17.1–19.8]	3116
Parity						
0	21.1 [18.9–23.5]	16.0 [14.1–18.2]	14.5 [12.7–16.6]	8.9 [7.5–10.7]	**5.6** [4.4–7.0]	1200
1	43.0 [40.7–45.3]	49.9 [47.5–52.2]	45.2 [42.8–47.4]	21.9 [20.0–23.8]	**23.3** [21.3–25.3]	1723
2	61.3 [59.5–63.1]	61.3 [59.5–63.1]	65.2 [63.5–67.0]	38.4 [36.6–40.2]	**26.8** [25.3–28.5]	2852
3	63.1 [61.2–65.0]	64.9 [63.0–66.8]	68.5 [66.7–70.3]	44.4 [42.4–46.3]	**24.1** [22.5–25.9]	2554
4 +	57.0 [55.5–58.6]	66.9 [65.3–68.3]	64.0 [62.4–65.4]	37.1 [35.5–38.6]	**26.9** [25.5–28.3]	3871
Education of women						
< 5	50.1 [48.7–51.4]	61.5 [60.2–62.8]	57.8 [56.5–59.1]	33.6 [32.4–34.9]	**24.2** [23.1–25.4]	5374
5–9	53.3 [51.5–55.0]	56.4 [54.7–58.2]	56.6 [54.9–58.4]	33.1 [31.5–34.8]	**23.5** [22.1–25.1]	3214
10–11	56.4 [53.2–59.5]	53.8 [50.6–56.9]	56.4 [53.2–59.5]	32.6 [29.7–35.6]	**23.8** [21.2–26.6]	958
12 +	60.5 [58.6–62.3]	52.8 [51.0–54.7]	58.5 [56.6–60.3]	35.8 [33.9–37.5]	**22.7** [21.2–24.3]	2654
Education of husband						
< 5	49.3 [47.4–51.3]	59.4 [57.5–61.3]	54.4 [52.5–56.3]	32.5 [30.7–34.4]	**21.9** [20.3–23.6]	2402
5–9	52.4 [50.8–54.0]	59.0 [57.5–60.6]	57.4 [55.9–59.0]	33.1 [31.7–34.7]	**24.3** [23.0–25.7]	4006
10–11	52.8 [50.6–55.0]	59.6 [57.4–61.7]	58.8 [56.7–61.0]	33.4 [31.4–35.5]	**25.4** [23.6–27.4]	2008
12 +	58.5 [56.9–60.1]	54.0 [52.4–55.6]	59.1 [57.5–60.6]	35.8 [34.3–37.4]	**23.3** [21.9–24.6]	3784
Husband occupation						
Non-agricultural labour	52.2 [50.9–53.6]	59.4 [58.0–60.7]	56.7 [55.4–58.2]	32.7 [31.5–34.1]	**24.0** [22.8–25.2]	4969
Salaried	57.2 [55.2–59.2]	54.0 [52.0–56.0]	54.7 [52.8–56.8]	35.3 [33.5–37.3]	**19.4** [17.9–21.1]	2352
Cultivator/ agricultural labour	51.2 [49.3–53.2]	57.1 [55.1–59.0]	59.2 [57.3–61.2]	34.2 [32.4–36.2]	**25.0** [23.3–26.7]	2599
Business	58.2 [56.0–60.3]	59.5 [57.3–61.7]	62.6 [60.3–64.6]	35.6 [33.4–37.7]	**27.0** [25.1–29.0]	1936
Other	43.7 [38.6–48.9]	51.9 [46.7–57.0]	47.9 [42.7–53.1]	27.8 [23.3–32.6]	**20.1** [16.3–24.6]	344
Fertility preference						
Want to have another child within 2 years	30.0 [27.7–32.4]	33.2 [30.9–35.7]	26.4 [24.3–28.8]	13.0 [11.4–14.9]	**13.4** [11.8–15.2]	1465
Want to have another child after 2 years	41.9 [39.5–44.4]	50.9 [48.4–53.4]	48.6 [46.2–51.2]	22.7 [20.7–24.9]	**25.9** [23.8–28.2]	1541
Want no more child	62.3 [61.3–63.3]	64.3 [63.3–65.3]	69.3 [68.3–70.3]	42.6 [41.6–43.7]	**26.7** [25.8–27.7]	8368
Desired sex composition						
No sex preference	44.8 [42.7–46.9]	48.3 [46.2–50.3]	49.2 [47.1–51.3]	27.3 [25.5–29.2]	**21.9** [20.3–23.7]	2251
Didn't achieve either sex	25.1 [22.6–27.8]	25.4 [22.8–28.1]	19.3 [17.0–21.8]	10.7 [8.9–12.7]	**8.6** [7.1–10.5]	1027
Achieved desired number of boys, but not girls	59.7 [57.5–61.9]	61.9 [59.7–64.1]	63.7 [61.5–65.8]	38.4 [36.2–40.6]	**25.3** [23.3–27.3]	1891
Achieved desired number of girls, but not boys	49.1 [47.1–51.2]	62.8 [60.8–64.8]	51.4 [49.3–53.4]	26.4 [24.6–28.2]	**25.0** [23.3–26.8]	2242
Achieved desired sex composition	64.1 [62.7–65.5]	65.0 [63.7–66.4]	70.5 [69.3–71.9]	44.0 [42.6–45.4]	**26.5** [25.3–27.8]	4789
FLW contacted on FP in last 3 years						
No	51.9 [50.9–52.9]	56.2 [55.2–57.1]	56.1 [55.2–57.1]	32.9 [32.0–33.8]	**23.2** [22.4–24.0]	10,493
Yes	65.1 [62.8–67.4]	67.2 [64.9–69.5]	66.5 [64.2–68.7]	40.0 [37.6–42.3]	**26.5** [24.5–28.7]	1707

Bold font is used to highlight traditional methods related estimates

19.4% were using rhythm method, 4.2% were using withdrawal method and 0.1% were using other traditional methods

In contrast to the modern contraceptive method, no significant differences were observed in the characteristics of TM users. For example, the TM use did not differ substantially by place of residence, age, caste, educational levels (self and husband) and parity. Except among the younger (19.3%) or women with parity 0 (5.6%), the use of the TM was almost consistent (about 23–29%) in all other age groups or parity.

### Initial contraceptive method and switching

**Fig. 1:**
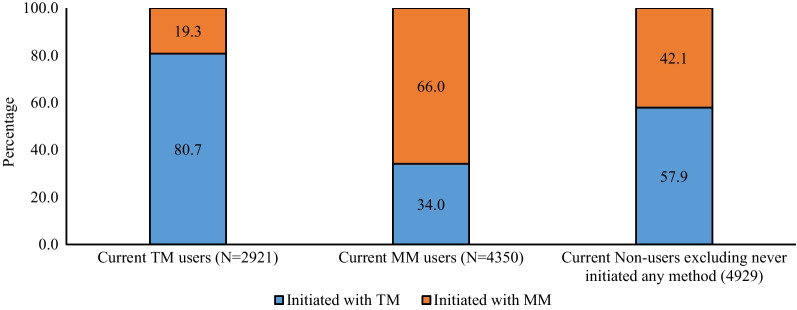
Percent distribution of current method users by initial contraceptive methods

Of 2921 current TM users, 80.7% reported that their initial contraceptive use was with TM, while the remainder initiated with MM (Fig. 1). This pattern was the same among MM users as well. Of 4350 current MM users, 66% reported that their initial contraceptive use was with MM while 34% initiated with TM. Among current non-users who ‘ever-used’ any contraceptive method, 57.9% reported that their initial contraceptive use was with TM and 42.1% initiated with MM.

**Fig. 2:**
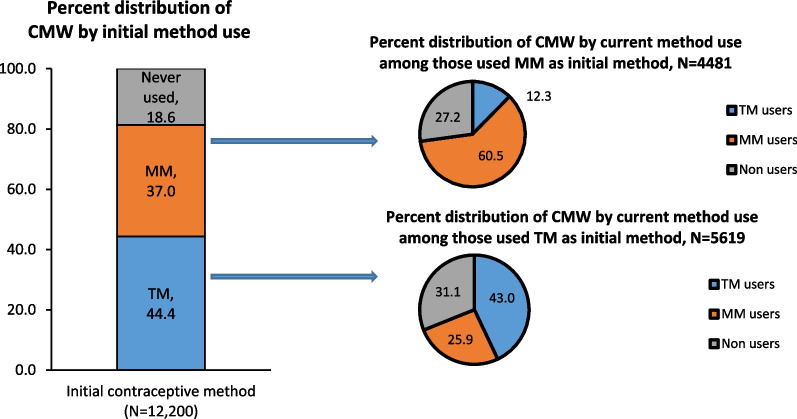
Percent distribution of CMW by switching from initial contraceptive method to current method

Overall, 44.4% of CMW initiated contraceptive use with TM, 37.0% initiated with MM, and 18.6% were lifetime non-users (Fig. 2). Initiation of TM use was higher among CMW belonging to rural areas, SC/ST and OBC, poorest wealth quintile households, with high parity, and less educated compared to their respective counterparts (Additional file 1: Table S2). Among those initiated with TM, 43.0% were currently using TM, 25.9% currently using MM and 31.1% not using any method at the time of the survey. Similarly, among those initiated with MM, most of the women (60.5%) were using MM, 12.3% currently using TM and 27.2% not using any method at the time of the survey.

### Consistency/switching in use of contraceptive methods and future intention

**Fig. 3:**
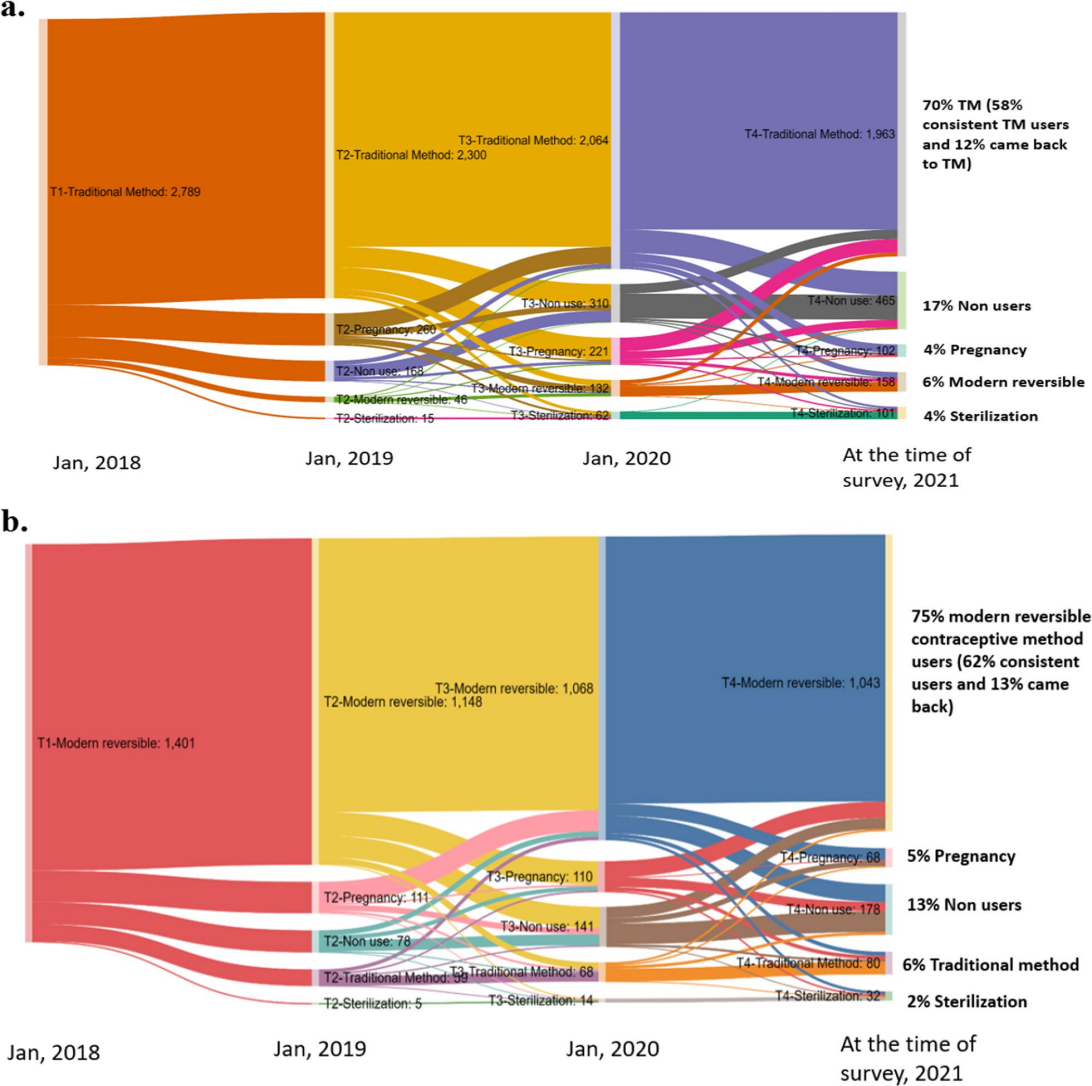
Three-year contraceptive use journey of a cohort of traditional users and modern reversible users (**a** TM users; **b** Modern reversible users)

**Fig. 4:**
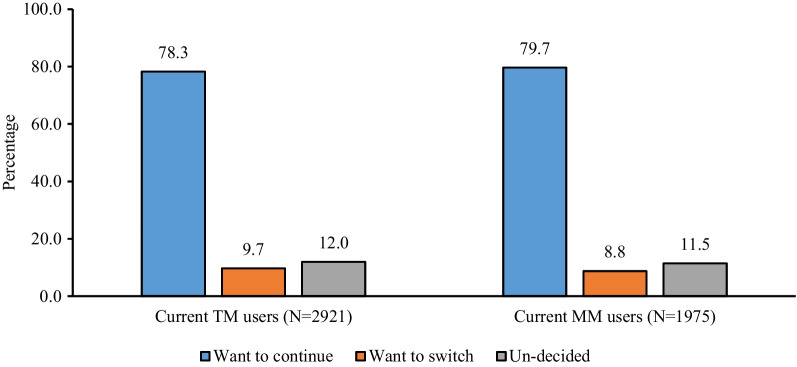
Percent distribution of CMW by their intention to continue with current method

Among 2796 cohort of TM users at the start of the calendar (January 2018), 59% were found to be using a TM consistently over the next 3 years (until the time of the survey) (Fig. 3a) whereas about 12% returned to TM as a method of their choice after either experiencing pregnancy, or were non-users for a certain period, or used any modern reversible methods. While about 16% became non-users, only 6% switched to any modern reversible methods, 4% opted for sterilization and 4% were pregnant at the time of the survey. Among 1401 modern reversible method users including IUCD, Injectables, Pills, and Condoms at the start of the calendar, 62% were consistent users, 13% came back to modern reversible method after other experiences, only 6% switched to TM, 2% opted for sterilization and 13% became non-users at the time of the survey (Fig. 3b). As far as the future intention of method use is concerned, 78.3% of current TM users and 79.7% of current MM users wanted to continue with the same method in the future (Fig. 4). Only 9.7% of current TM users and 8.8% of current MM users wanted to switch to another method while the remaining were indecisive about their future contraceptive use.

### Knowledge of contraceptive methods and reasons for continuing the current method

Analysis was conducted to understand whether there was a difference in the correct knowledge about the use of contraceptive methods among current TM users and MM users leading to a differential in the current choice and future preferences. Table 3 depicts that half of the current TM users and 45.5% of current MM users had the correct knowledge of the ovulatory cycle. Among the current TM users, 81.4% also had correct knowledge of condoms, 54.3% about IUCD, 23.0% on injectables, and 18.7% had correct knowledge of daily contraceptive pills. A similar pattern was observed among the current modern reversible method users as far as their correct knowledge about other modern methods was concerned. The majority of the TM users (78.4%) also reported that they received information on the TM from their peer groups.

**Table 3:** Percentage of CMW by their knowledge about methods, reasons for initiating and continuing the current method and their source of information by current method use

	Current TM users	Current MM users
Percent of CMW having correct knowledge about:		
Ovulatory cycle	50.2	45.4
Condom	81.4	86.7
IUCD	54.3	58.4
Injectables/Antara	23.0	25.0
Pills	18.7	20.9
ECP	9.9	16.0
Chhaya/Centchroman	1.3	1.5
Any modern reversible method (of the above six methods)	87.9	91.9
N	2921	4350
Reasons for continuing the current method		
No side effects	56.9	53.6
Easy to use	41.7	46.4
No cost incurred	38.0	12.2
Highly effective in preventing pregnancy	30.8	40.4
Easily available	27.6	47.4
Infrequent sex	10.3	5.9
No religious prohibition	4.8	4.2
Not to worry about running out of stock	3.6	2.2
Others	1.5	1.1
N	2264	1544
Source of information about the current method (multiple responses)		–
Peer groups (husband/ friends/ neighbours)	78.4	
Public health facility	1.3	
Private health facility	1.4	
Media (internet/ newspaper)	2.5	
Others	7.6	
Nowhere	12.5	
N	2921	

The predominant reasons reported for continuing current TM use included no side effects (56.9%%), easy to use (41.7%), no cost involved (38.0%), and easy availability (27.6%). Similarly, the major reasons for using the current method by MM users were no side effects (53.6%), easy availability (47.4%), ease to use (46.4%), and highly effective in preventing pregnancy (40.4%). ‘Infrequent-sex’, “no religious prohibition” and “not to worry about running out of stock” were the least reported reasons both by TM users and MM users.

### Availability of family planning methods in public health facilities

Table 4 presents the change in the availability of FP services and commodities in 289 public health facilities between 2018 and 2021. In 2021, 81.3% of these public health facilities had three or more FP commodities against 66.1% in 2018. Importantly, the mean number of FP commodities available in public health facilities increased from 3.5 in 2018 to 4.7 in 2021. The availability of two newly introduced contraceptive methods also increased considerably during this period with injectables/*Antara* from 17.0% in 2018 to 78.2% in 2021 and *Chhaya* from 16.3% in 2018 to 63.7% in 2021. We also found that 84% of public health facilities had sterilization services available which remained unchanged since 2018.

**Table 4:** Availability of modern reversible contraceptive methods in public health facilities

	2018	2021
% of public health facilities with availability of LAP/Mini-LAP/NSV services	84.4	84.1
% of public health facilities with availability of modern reversible contraceptive methods:		
IUCD-375/ 380-A	72.0	80.3
Injectable/Antara	17.0	78.2
Condom	73.4	77.5
ECP	57.8	73.7
Pills	64.7	72.3
Chhaya/Centchroman	16.3	63.7
% of public health facilities with availability of number of modern reversible contraceptive methods		
1 or more	81.7	92.4
2 or more	73.4	87.5
3 or more	66.1	81.3
4 or more	56.4	76.5
5 or more	13.1	65.7
All 6	10.4	42.2
Mean number of modern contraceptive methods available in public health facilities *(minimum–maximum)*	3.5 (*0–6*)	4.7 (*0—6*)
Number of public health facilities	289	289

2018—UPTSU facility mapping data; 2021—Facility assessment data from integrated FP survey

### Past contraceptive use and future intention among CMW with unmet need

Table 5 depicted that of 1746 CMW with unmet need, 33.3% had ever used any modern method while 42.6% has ever used any traditional method. One-third of the CMW with unmet need initiated with a modern method, 26.3% initiated with a traditional method and 40.9% never used any method. Overall, 36.7% of the CMW with unmet need reported that they intended to use a contraceptive method in the next one year. Among those intending to use a method in the next one year, 28.7% intent to use TM, 26.5% intent to use condoms, 23.9% intent to undergo sterilization, and less than 10% intent to use other modern reversible methods.

**Table 5:** Percentage of CMW with unmet need contraceptive method ever used, initial method and intention to use any method in next 1 year

	Percentage
Ever used any MM (N = 1746)	33.3
Ever used any TM (N = 1746)	42.6
Initial method (N = 1746)	
MM	32.8
TM	26.3
Never used any method	40.9
Intent to use any method in the next 1 year (N = 1746)	
Yes	36.7
No	40.9
Don’t know	22.4
Types of methods intended to use in the next 1 year (N = 689)	
TM	28.7
Condom	26.5
Sterilization	23.9
Injectable/Antara	9.6
Pills	7.6
IUCD	3.7
Chhaya/ Centchroman	3.1
ECP	0.2
Other modern methods	0.5

## Discussion

Even though traditional methods are relatively less effective compared to modern contraceptive methods [19], the use of traditional methods have continued to increase over time in UP. Traditional methods were used by one-fourth of CMW followed by sterilization (17.5%), condoms (12.6%) and other modern methods (3.9%). We found that the current method was linked to their initial method use and a majority of CMW continued with the same method for at least three-year period preceding the survey. The initiation of TM was higher among CMW belonging to rural areas, SC/ST and OBC category, poorest household, higher parity, and less educated women. We also found that the majority of the CMW reported an intention to continue their current method in future as well.

While it has been hypothesized that the low availability and accessibility of modern contraceptives might have played role in the increased use of TM in UP [12], our analysis showed that the overall availability of family planning methods has increased in public health facilities in UP, yet a large proportion of women chose TM. Again, contrary to the evidence that the lack of knowledge of modern methods [20] or the fertile period [21] impedes its usage, we found that a substantial proportion of CMW using TM had correct knowledge of modern methods, especially about condoms which is the most common modern reversible method in UP as well as the fertile period. The above findings indicate that TM usage in UP could be a preference rather than due to unavailability of modern methods or lack of knowledge about other contraceptive methods. This finding was also supported by the findings on reasons for preferring TM which included—the convenience of use of TM, no side effects, no cost incurred etc. Similar reasons were also found in other studies as reasons for using TM over the MM [22–24].

Earlier studies on TM in the Indian context showed a higher likelihood of TM use among older women, highly educated, rural, non-poor women, having 2 plus living children [11]. However, in this study, no major differences were observed which again indicated that the increase as well as high usage of TM among CMW is likely to be due to preference rather than differentials in socio-demographic and economic positions of women. In contrast, our findings on differentials in the use of the modern contraceptive methods by socio-demographic characteristics of CMW corroborates with other studies that demonstrated higher use of modern contraceptives among the CMW belonged to urban areas, Hindu religion, richest wealth quintile, older age group and higher parity compared to their respective counterparts [25].

One of the important findings of this study was that the initial contraceptive method used has a larger implication on current method use, consistency and continuation of the same method. Existing studies on family planning have placed limited attention on understanding the role of initial contraceptive method use on the current method. For instance, among the current traditional method users, 80.7% started with the TM, and among the current modern contraceptive method users, 66.0% started with a MM. Similarly, of those who started with the TM, over two-fifths (43.0%) were using the TM, as well of those who started with the MM, 60.5% still used the modern method. Further, we found that TM usage was consistent for at least a three-year period before the survey among a substantial proportion of CMW as documented elsewhere [26]. Same analysis disaggregated by age groups (15–24, 25–29 and 30 + years) showed that younger women had less overall consistency of TM use over three-year period as compared to 30 + aged women (Additional file 1: Fig. S1). However, the low consistency in the younger age group was not converted to MM use in the three-year period for which calendar data was collected. The conversion to modern reversible or limiting method for the TM users in three age groups was only 12%, 16%,  7%, respectively, indicating the importance of initial method. Moreover, four-fifths of the current TM users showed their intention to continue using TM in future as well. These findings have significant programmatic relevance as they highlight the importance of reaching young couples soon after marriage and introducing a more effective modern family planning method as the initial method of choice.

Lastly, our analysis of initiation and future contraceptive use among CMW with unmet need indicated no major differences in the method they initiated or ever used, but a considerable proportion of them intend to use TM in future followed by condom and sterilization. This finding also has programmatic implications for immediate further reduction of unmet need through modern methods.

Uttar Pradesh has more than 150,000 ASHAs who can potentially reach the newlyweds at the earliest through community outreach in their catchment area to initiate modern family planning method choices. They can be empowered with supplies of appropriate contraceptive commodities, training them in counselling related to family planning so that correct information regarding different methods of contraception, their effectiveness, side-effects and avenues to reach out for their requirement could be informed to the newlyweds. Availability of contraceptive methods can be taken closer to the community for better adoption by using well-established platforms like VHND in UP. According to NFHS-5, institutional delivery in UP has increased to 83.4% with 57% delivered in public health facilities. This provides a potential programmatic opportunity to counsel and initiate post-pregnancy modern family planning choices through trained providers. VHNDs can also be used to provide family planning services to mothers coming for the immunization of their children. To cater to the CMW having unmet need for limiting method, the program should augment the provider base for sterilization services, convert fixed-day services to regular services, and ensure provision of services throughout the year. These programmatic measures together would help in achieving the sustainable development goal of 75% demand satisfied by modern contraceptive methods.

The current study has a few limitations as well. Firstly, the study used retrospective calendar data on method use and fertility events for three years to analyze the continuity of method used. Any recall bias might have affected the results. However, we do not expect a major recall bias as women were able to answer the events over time, otherwise would have expected many responses of “Don’t know” or “No answer”. Secondly, the study was cross-sectional and not prospective, limiting the ability to understand unintended pregnancy, abortion etc. among different method users. Thirdly, the availability of modern contraceptive methods in private health facilities and pharmacies was not collected. Lastly, the contraceptive calendar data didn’t have information on the wantedness of pregnancy for each pregnancy that occurred during the last 3 years but only for the last pregnancy.

## Conclusion

Demand for contraceptive methods and use of TM has been increasing in UP. This study found one-fourth of the CMW were using TM and it was popular across most population subgroups, even with increased availability of free modern contraceptive methods in public health facilities. Current TM users as well as CMW with unmet need also expressed a future preference to use TM. The initial contraceptive method seems to have significant implications for current contraceptive method use as well as future preferences, necessitating the program planners to reach the young and low parity women with modern method choice. Reaching out to the young couples through the vast network of front-line workers in rural UP, augmenting and skill-building of community and facility level providers of modern methods could be a potential pathway. More intense policy discussions on recognizing TM use as a preferred choice are required while fixing up national and state-level targets beyond modern contraceptive usage.

## Supplementary Information

**Additional file 1: Table S1.** Method-specific definition of correct knowledge. **Table S2.** Percentage of CMW by their initial contraceptive method according to socio-demographics. **Fig. S1.** Three-year contraceptive use journey of a cohort of traditional users (a. TM users—15–24 years; b. TM users—25–29 years; c. TM users—30 + years)

## Data Availability

The data that support the findings of this study are available from the corresponding author upon reasonable request.

## Abbreviations

ANM: Auxiliary Nurse Midwives
ASHA: Accredited Social Health Activist
CEB: Census Enumeration Blocks
CMW: Currently Married Women
FLW: Front Line Workers
FP: Family Planning
GoUP: Government of Uttar Pradesh
ICPD: International Conference on Population and Development
IHAT: India Health Action Trust
mCPR: Modern Contraceptive Prevalence Rate
MDG: Millennium Development Goals
MM: Modern Methods
NFHS: National Family Health Surveys
OBC: Other Backward Castes
ODK: Open Data Kit
PPS: Probability Proportional to Size
PSU: Primary Sampling Units
SC: Scheduled Caste
SD: Standard Deviation
SDG: Sustainable Development Goals
ST: Scheduled Tribe
TM: Traditional Methods
UP: Uttar Pradesh
UPTSU: Uttar Pradesh Technical Support Unit
VHND: Village Health and Nutrition Day
