# Supplementary material for: Understanding the rise in traditional contraceptive methods use in Uttar Pradesh, India
Source: Reprod Health. 2023 Jan 6;20:8. doi: 10.1186/s12978-022-01547-y (PMC9817250; doi:10.1186/s12978-022-01547-y)

**Additional file 1: Table S1. Method-specific definition of correct knowledge**

| ***Contraceptive Methods*** | **Correct knowledge definition** |
| --- | --- |
| *Condom* | If the response was ‘once’ to the question *“According to you how many times a condom can be used?”* |
| *IUCD* | If the response was 5 or 10 years to the question *“According to you, for how many months pregnancy can be averted from IUCD?”* |
| *Injectables/ Antara* | If the response was ‘3 months’ to the question *“After how many months, the dose of Antara has to be repeated?”* |
| *Pills* | If the response was ‘take two pills next day’ to the question “*If a woman is using Mala- D or N and she forgets to take her pill one day, then what should she do?”* |
| *ECP* | If the response was ‘72 hours’ to the question *“Within how many hours of unprotected sexual intercourse should a woman take an ECP?”* |
| *Chhaya/ Centchroman* | If the response was ‘twice a week for the first 3 months followed by once a week from 4^th^ month onwards’ to the question “*If a woman is willing to start Chhaya, what is the frequency of taking these pills?”* |
| *Ovulatory cycle* | If repose was ‘halfway between two periods (i.e, between  10-20 days’ to the question “*According to you what is the most unsafe period for conception among those women who has regular periods?”* |

**Additional file 1: Table S2. Percentage of CMW by their initial contraceptive method according to socio-demographics**

| Characteristics | Tm | Modern | Neverused | Total | N |
| --- | --- | --- | --- | --- | --- |
| Overall | **44.4** | **37.0** | **18.6** | **100.0** | **12200** |
| Place of residence |  |  |  |  |  |
| *Rural* | 46.9 | 33.9 | 19.2 | 100.0 | 9,704 |
| *Urban* | 36.1 | 47.2 | 16.6 | 100.0 | 2,496 |
| Caste |  |  |  |  |  |
| *SC/ST* | 47.6 | 33.8 | 18.6 | 100.0 | 3,665 |
| *OBC* | 45.1 | 36.2 | 18.7 | 100.0 | 6,438 |
| *Others* | 37.6 | 44.7 | 17.7 | 100.0 | 2,041 |
| Religion |  |  |  |  |  |
| *Hindu* | 45.2 | 37.2 | 17.6 | 100.0 | 10,369 |
| *Non-Hindu* | 40.6 | 35.8 | 23.6 | 100.0 | 1,790 |
| Wealth quintile |  |  |  |  |  |
| *Poorest* | 49.4 | 29.4 | 21.2 | 100.0 | 2,028 |
| *Poor* | 48.8 | 31.3 | 19.8 | 100.0 | 2,457 |
| *Middle* | 46.5 | 34.7 | 18.8 | 100.0 | 2,622 |
| *Rich* | 42.5 | 40.1 | 17.4 | 100.0 | 2,609 |
| *Richest* | 36.1 | 48.0 | 15.8 | 100.0 | 2,443 |
| Current age |  |  |  |  |  |
| *15-24* | 34.1 | 28.2 | 37.7 | 100.0 | 2,556 |
| *25-29* | 43.2 | 41.3 | 15.5 | 100.0 | 2,508 |
| *30-34* | 47.1 | 40.4 | 12.5 | 100.0 | 2,145 |
| *35-39* | 45.8 | 42.0 | 12.3 | 100.0 | 1,875 |
| *40-49* | 51.1 | 35.1 | 13.8 | 100.0 | 3,116 |
| Parity |  |  |  |  |  |
| *0* | 13.0 | 20.3 | 66.7 | 100.0 | 1,200 |
| *1* | 36.0 | 36.3 | 27.7 | 100.0 | 1,723 |
| *2* | 43.6 | 44.8 | 11.6 | 100.0 | 2,852 |
| *3* | 51.1 | 39.4 | 9.5 | 100.0 | 2,554 |
| *4+* | 54.5 | 35.1 | 10.4 | 100.0 | 3,871 |
| Education of women |  |  |  |  |  |
| *<5* | 51.3 | 31.1 | 17.6 | 100.0 | 5,374 |
| *5-9std* | 42.8 | 36.7 | 20.5 | 100.0 | 3,214 |
| *10-11* | 39.3 | 40.8 | 19.9 | 100.0 | 958 |
| *12+* | 34.5 | 47.5 | 18.0 | 100.0 | 2,654 |
| Education of husband |  |  |  |  |  |
| *<5* | 50.0 | 30.5 | 19.5 | 100.0 | 2,402 |
| *5-9std* | 46.9 | 34.5 | 18.7 | 100.0 | 4,006 |
| *10-11* | 46.1 | 35.9 | 18.0 | 100.0 | 2,008 |
| *12+* | 37.3 | 44.5 | 18.2 | 100.0 | 3,784 |

**Additional file 1: Fig. S1. Three-year contraceptive use journey of a cohort of traditional users and 3modern reversible users**

**Fig S1.a. TM users – 15-24 years**


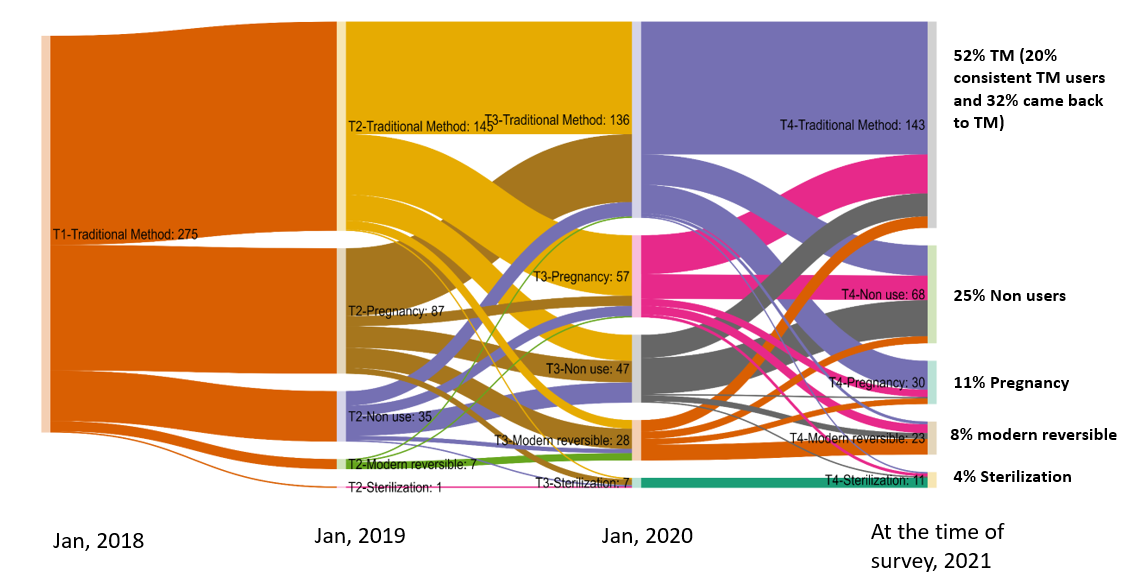


**Fig S1.b.**
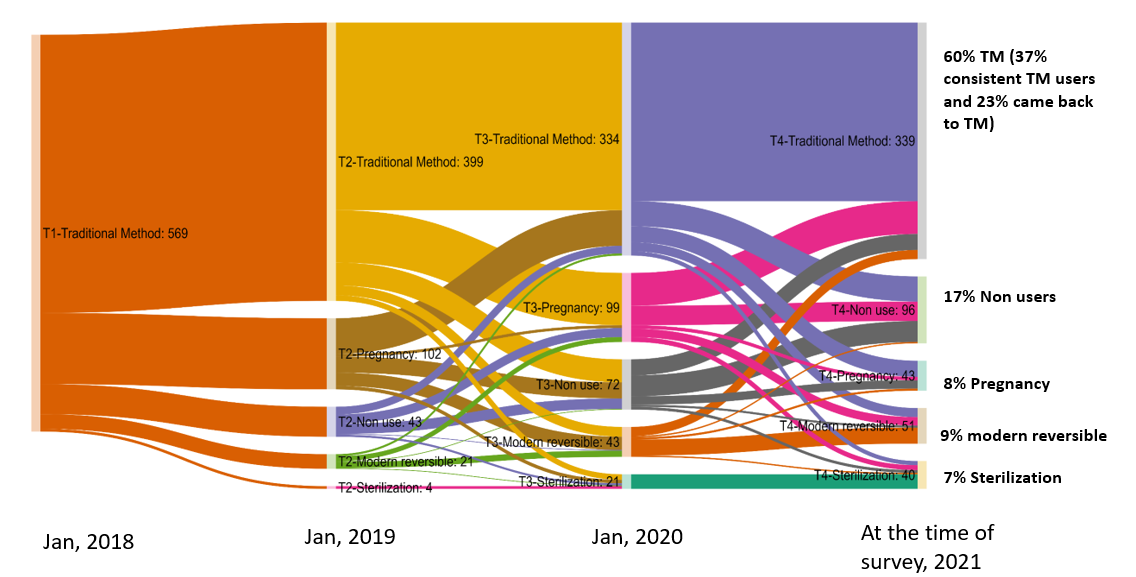
**TM users – 25-29 years**

**Fig S1.c. TM users – 30+ years**


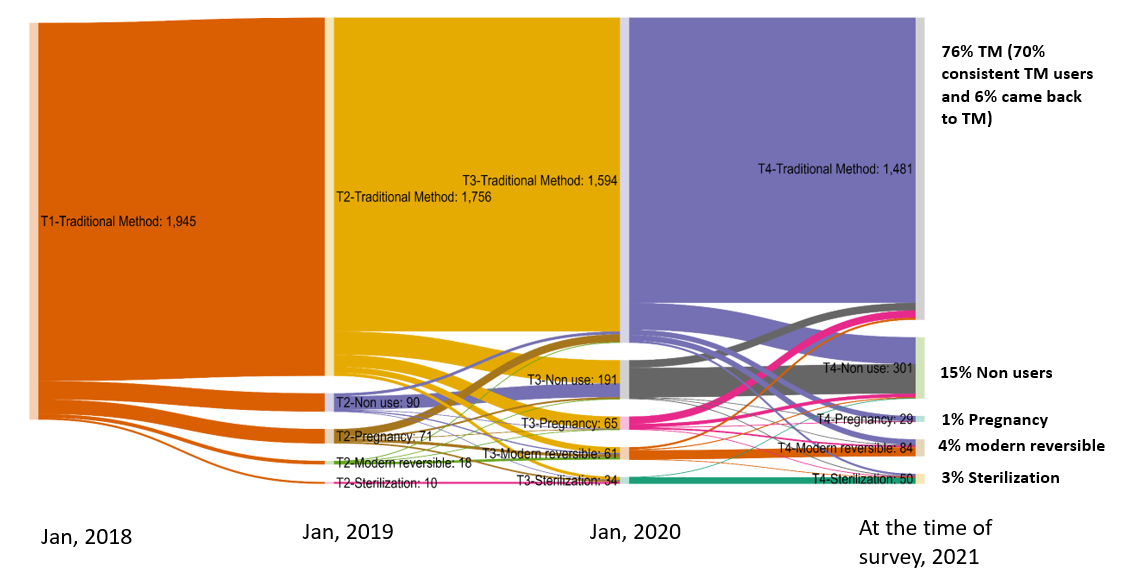

Supplement: Supplementary file 1 — Additional file 1: Table S1. Method-specific definition of correct knowledge. Table S2. Percentage of CMW by their initial contraceptive method according to socio-demographics. Fig. S1. Three-year contraceptive use journey of a cohort of traditional users (a. TM users—15–24 years; b. TM users—25–29 years; c. TM users—30 + years) [file 12978_2022_1547_MOESM1_ESM.docx]
